# Susceptibility of *Pediococcus *isolates to antimicrobial compounds in relation to hop-resistance and beer-spoilage

**DOI:** 10.1186/1471-2180-9-190

**Published:** 2009-09-07

**Authors:** Monique Haakensen, David M Vickers, Barry Ziola

**Affiliations:** 1 Department of Pathology and Laboratory Medicine, Room 2841 Royal University Hospital, 103 Hospital Drive, University of Saskatchewan, Saskatoon, SK S7N 0W8, Canada; 2 Department of Computer Science, 110 Science Place, University of Saskatchewan, Saskatoon, SK, S7N 5C9, Canada

## Abstract

**Background:**

Though important in the context of food microbiology and as potential pathogens in immuno-compromised humans, bacterial isolates belonging to the genus *Pediococcus *are best known for their association with contamination of ethanol fermentation processes (beer, wine, or fuel ethanol). Use of antimicrobial compounds (e.g., hop-compounds, Penicillin) by some industries to combat *Pediococcus *contaminants is long-standing, yet knowledge about the resistance of pediococci to antimicrobial agents is minimal. Here we examined *Pediococcus *isolates to determine whether antibiotic resistance is associated with resistance to hops, presence of genes known to correlate with beer spoilage, or with ability to grow in beer.

**Results:**

Lactic acid bacteria susceptibility test broth medium (LSM) used in combination with commercially available GPN3F antimicrobial susceptibility plates was an effective method for assessing antimicrobial susceptibility of *Pediococcus *isolates. We report the finding of Vancomycin-susceptible *Pediococcus *isolates from four species. Interestingly, we found that hop-resistant, beer-spoilage, and beer-spoilage gene-harbouring isolates had a tendency to be more susceptible, rather than more resistant, to antimicrobial compounds.

**Conclusion:**

Our findings indicate that the mechanisms involved in conferring hop-resistance or ability to spoil beer by *Pediococcus *isolates are not associated with resistance to antibiotics commonly used for treatment of human infections. Also, Vancomycin-resistance was found to be isolate-specific and not intrinsic to the genus as previously believed.

## Background

Isolates from the genus *Pediococcus *are particularly problematic for the brewing industry where hop-compounds are used to provide flavour to beer. Hop-compounds are antimicrobial in that they dissipate the trans-membrane pH gradient of microbes, thereby inhibiting growth and potential spoilage of product [1]. As pediococci are also used as beneficial microbes in the context of food microbiology and animal husbandry (e.g., wine, cheese, and yogurt industries as well as for the production of silage), the emergence of hop-resistant *Pediococcus *isolates in the brewing industry is of broader interest. These isolates frequently harbour one or more ATP-binding cassette type multidrug resistance (ABC MDR) genes, suggesting that resistance to hop-compounds may also confer resistance to other antimicrobial compounds [2]. We have previously shown that several genes can be correlated with ability of *Pediococcus *isolates to grow in beer and to resist the antimicrobial activity of hop-compounds [3-5]. These are the ABC MDR genes *ABC2*, *bsrA*, *bsrB*, [6] and *horA *[2], a putative divalent cation transporter known as *hitA *[7], and *horC *which codes for a protein possessing little homology to any known protein [8,9].

Because, many pediococci possess special growth requirements, conventional antimicrobial-sensitivity testing media have been demonstrated to be unsuitable for testing of *Pediococcus *isolates for antimicrobial resistance [10-12]. However, enriched media that permits growth of pediococci may inhibit the antimicrobial activity of some compounds under investigation. Previously, antimicrobial susceptibility testing of *Pediococcus *isolates has been attempted by several methods, many of which are performed using some variety of agar diffusion [10,11,13,14]. However, more recently, dilution methods have been preferred over diffusion tests as the former allow for determination of minimum inhibitory concentrations (MICs), which is a more reliable and reproducible indicator of resistance [10,11]. For these reasons, lactic acid bacteria susceptibility test broth medium (LSM), which was recently developed by Klare *et al. *[11], should be considered the new testing standard for assessing the antimicrobial resistance spectra of lactic acid bacteria. Despite this medium being shown to be very effective for establishing antimicrobial susceptibilities of two species of *Pediococcus*, namely, *P. acidilactici*, and *P. pentosaceus *[10], it previously has not been used to study the prevalence, and spectrum, of antimicrobial resistance among other members of the genus.

Overall, the use of antimicrobial compounds by industries such as animal husbandry, brewing, and fuel ethanol to combat *Pediococcus *contaminants (e.g., hop-compounds, Penicillin, and Virginiamycin which is structurally similar to Synercid) is long-standing. However, knowledge about the resistance of pediococci to antimicrobial agents is minimal [12]. As such, the focus of this research was to determine whether the use of antimicrobial hop-compounds in the brewing industry is associated with an increase in the overall antimicrobial resistance of *Pediococcus *isolates. Here we report on the testing of isolates from six species of the genus *Pediococcus *against 17 antimicrobial compounds using LSM broth in commercially available Sensititre GPN3F Gram-positive MIC plates (TREK Diagnostic Systems, Cleveland OH).

## Results

### Antimicrobial susceptibility testing

Twenty-nine isolates, including six species of the *Pediococcus *genus were tested. Distribution of isolates by species and their ability to grow in beer is given in Table 1. Antimicrobial resistance testing was reproducible and the LSM by itself (containing no antimicrobial compounds) was permissive to the rapid growth of all *Pediococcus *isolates tested. All isolates used in this study were capable of producing visible turbidity in LSM broth after an incubation period of 24 hours. Isolates were cultured for a period of 48 hours in GPN3F plates so as to allow formation of larger bacterial pellets and thus a more accurate determination of the MIC for a given antibiotic. All control wells in the GPN3F plates produced appropriate results. Eight of the 29 isolates were randomly selected and tested in duplicate by the same method, and no variance in MICs was observed. The antimicrobial compounds and dilutions tested by the GPN3F antimicrobial susceptibility plates are listed in Additional file 1.

**Table 1:** *Pediococcus *isolates.

Species	N	Origin			Growth in Beer^a^	
			
		Brewery	Other^b^	Unknown	+	-
*acidilactici*	6	4	1	1	1	5
*claussenii*	12	12	0	0	11	1
ropy^c^	(5)	(5)	(0)	(0)	(5)	(0)
non-ropy^d^	(7)	(7)	(0)	(0)	(6)	(1)
*damnosus*	1	1	0	0	0	1
*inopinatus*	1	1	0	0	0	1
*parvulus*	5	0	5	0	1	4
ropy	(1)	(0)	(1)	(0)	(0)	(1)
non-ropy	(4)	(0)	(4)	(0)	(1)	(3)
*pentosaceus*	4	1	2	1	0	4
**Total**	**29**	**19**	**8**	**2**	**13**	**16**

^a ^Previously reported by Haakensen *et al. *[3,4].

^b ^Isolates of known non-brewery origin, specific origins are provided in Additional file 2.

^c, d ^Isolates positive and negative for exopolysaccharide rope production, respectively.

### Distribution of MIC by species, isolate, and ropy phenotype

Resistance to the 17 antimicrobial compounds and hop-compounds was determined, and the antimicrobial compounds to which resistant isolates of *Pediococcus *were found are given in Additional file 1. For the majority of the 29 isolates tested, a moderate degree of susceptibility was shown to each of the antibiotics and a MIC value could be determined. However, for two of the antibiotics (i.e., Vancomycin and Ciprofloxacin), the majority of isolates (72% and 52%, respectively) grew in the presence of the antibiotic at all concentrations tested. Additionally, 48% of isolates were hop-resistant. When *Pediococcus claussenii *and *Pediococcus parvulus *were assessed on the basis of ropy (i.e., exopolysaccharide-producing) phenotype, there was no significant difference found among the MICs for each antibiotic [Additional files 1 and 2].

Analysis of antimicrobial resistance according to *Pediococcus *species demonstrated that just over half of the antibiotics (9/17) had significantly different MICs for different species (Table 2 and Additional files 1 and 2). The non-parametric Kruskal-Wallis *H*-test was used to test for equality in population medians. This test is an extension of the Mann-Whitney *U*-test which is designed to examine whether two samples of observations come from the same distribution. Unfortunately, *post-hoc *analyses to determine which of the six species had significantly different MICs for each antibiotic was not possible due to the low number of isolates per species. However, when *P. claussenii *isolates were compared to isolates of the other species combined, *P. claussenii *had significantly lower MICs (Mann-Whitney *U*-test, *p *< 0.05) for all antimicrobial compounds tested, except for Erythromycin, Clindamycin, Daptomycin, and Vancomycin (data not shown).

**Table 2:** Antimicrobial compounds having significantly different MICs among the six *Pediococcus *species.

Antimicrobial compound	*p*-value^a^
Ampicillin	< 0.02
Ceftriaxone	< 0.02
Ciprofloxacin	< 0.02
Daptomycin	< 0.02
Gatifloxacin	< 0.01
Gentamicin	< 0.05
Levofloxacin	< 0.01
Penicillin	< 0.02
Synercid	< 0.05

^a ^*p*-value corresponds to the *H*-test statistic as derived from the non-parametric Kruskal-Wallis *H*-test which tests for equality in population medians where there are three or more groups.

### Distribution of MIC by presence of genes associated with beer-spoilage and/or hop-resistance

Whether any of the beer-spoilage and/or hop resistance-correlated genes *ABC2*, *bsrA, bsrB, hitA, horA*, and *horC *were associated with any of the antimicrobial MICs was determined [Additional file 2]. Of these six genes, *hitA, horC*, and *ABC2*, did not occur with sufficient frequency to be analyzed statistically. The *bsrA*, *bsrB*, and *horA *genes unexpectedly demonstrated significant associations not with antibiotic resistance, but with susceptibility to antimicrobial compounds (*bsrA *and *bsrB *with Ampicillin, Levofloxacin, Penicillin, Ciprofloxacin, Gatifloxacin, and Oxacillin + 2% NaCl; *horA *with Erythromycin).

### Distribution of MIC by hop-resistance phenotype

Fourteen of the 29 isolates (48.3%) were deemed resistant to hop-compounds as tested by the hop-gradient agar plate with ethanol method. When the isolates categorized according to susceptibility or resistance to hop-compounds had their MICs compared using the Mann-Whitney *U*-test, 29.4% (5/17) of the antimicrobial compounds had significantly lower MICs for the hop-resistant isolates (Table 3). Of these five antimicrobials, only Ciprofloxacin showed a significant correlation with hop-resistance. Unexpectedly, the correlation was a negative one (Spearman's *ρ *= -0.47, *p *< 0.01), since as the MIC for Ciprofloxacin increased, the probability of an isolate's growth in the presence of hop-compounds decreased.

**Table 3:** Antimicrobial compounds having significantly lower MICs in hop-resistant isolates^a^.

Antimicrobial compound	Median and Distribution of MIC (μg/ml)		*p*-value^b^
		
	Hop-resistant	Hop-sensitive	
Ampicillin	0.25 (0.12-4)	1 (0.12-4)	< 0.05
Ciprofloxacin	2 (0.5-NR^c^)	4 (0.5-NR)	< 0.05
Gatifloxacin	1 (0.5-8)	4 (1-NR)	< 0.05
Penicillin	0.12 (0.06-NR)	2 (0.06-NR)	< 0.02
Rifampin	0.5 (0.5-2)	1 (0.5-NR)	< 0.05

^a ^Hop-resistance is as determined by the hop-gradient agar plate with ethanol method.

^b ^*p*-value corresponds to *U*-test statistic as derived from the non-parametric Mann-Whitney *U*-test which is designed to examine whether two samples of observations came from the same distribution.

^c ^NR; MIC not reached, isolate could grow at highest concentration of antibiotic tested.

### Distribution of MIC by ability to grow in beer

Of the 29 *Pediococcus *isolates tested, 13 (44.8%) were capable of growing in beer. The results of testing for an association between antibiotic susceptibility and growth in beer are given in Table 4. Based on a Mann-Whitney *U*-test, eight of the 17 antibiotics tested demonstrated a significantly *lower *MIC in those isolates that could grow in beer.

**Table 4:** Antimicrobial compounds having significantly lower MICs in isolates able to grow in beer.

Antimicrobial compound	Median and Distribution of MIC (μg/ml)		*p*-value^a^
		
	Grow in Beer	Cannot grow in beer	
Ampicillin	0.25 (0.12-4)	2 (0.12-4)	< 0.01
Ciprofloxacin	2 (0.5-NR^b^)	4 (0.5-NR)	< 0.01
Gatifloxacin	1 (0.25-8)	4 (1-NR)	< 0.01
Levofloxacin	2 (0.5-NR)	16 (1-NR)	< 0.05
Oxacillin + 2% NaCl	0.25 (0.25-4)	1 (0.25-NR)	< 0.02
Penicillin	0.12 (0.12-NR)	1 (0.06-NR)	< 0.01
Synercid	0.5 (0.12-1)	1 (0.25-2)	< 0.05
Trimethoprim/Sulfamethoxazole	0.5/9.5 (0.5/9.5-NR)	R (0.5/9.5-NR)	< 0.05

^a^*p*-value corresponds to *U*-test statistic as derived from the non-parametric Mann-Whitney *U*-test which is designed to examine whether two samples of observations came from the same distribution.

^b ^NR; MIC not reached, isolate could grow at highest concentration of antibiotic tested.

## Discussion

The finding of *Pediococcus *isolates that showed only moderate resistance to Vancomycin is discordant with other studies to date which have consistently reported the genus *Pediococcus *to be intrinsically Vancomycin-resistant [10,12-14]. The isolates that were not resistant to all concentrations of Vancomycin tested were from the species *P. acidilactici *(N = 1), *P. claussenii *(Ropy, N = 1; Non-ropy, N = 3), *P. damnosus *(N = 1), and *P. parvulus *(Non-ropy, N = 2), suggesting that the phenomenon is not the product of a clonal event. It has previously been shown that intrinsic Vancomycin resistance in *P. pentosaceus *is due to a modified peptidoglycan precursor ending in D-Ala-D-lactate [15]. While this may also be the mechanism used by other Vancomycin-resistant pediococci, it is likely that the eight susceptible isolates do not possess this mechanism. Because media previously used for *Pediococcus *antimicrobial susceptibility testing have since been shown to be inappropriate for such testing (11), it is possible that the earlier finding of intrinsic *Pediococcus *Vancomycin-resistance was an artifact of the testing medium used, rather than reflective of pediococci genetic content.

The ropy phenotype did not associate with resistance to any of the antimicrobial compounds tested. This was an unexpected result as the ropy phenotype acts to create a biofilm which is expected to act as a physical barrier for the bacteria, putatively protecting them from the antimicrobial compounds. Why no associations were found is unclear. It may be that the type of exopolysaccharide matrix produced by these isolates did not result in a sufficiently dense matrix so as to inhibit the passage of antimicrobial compounds. Alternatively, the amount of energy expended on the production of exopolysaccharide may have caused a decreased ability to grow in the presence of the antimicrobial compounds, despite the partial antimicrobial barrier created by the exopolysaccharide.

Of particular interest to the brewing industry is the presence in pediococci of hop-resistance or beer-spoilage correlated genes (*ABC2*, *bsrA, bsrB, hitA, horA*, and *horC*). Of these six genes, only *horA *has been conclusively shown to function as a multidrug transporter, however, the *ABC2, bsrA*, and *bsrB *genes are highly similar to known ABC MDR genes, and the *hitA *gene is similar to divalent cation transporters. As such, all six of these beer-spoilage or hop-resistance correlated genes were assessed for associations with antimicrobial resistance. The genes *hitA, horC*, and *ABC2 *did not occur with sufficient frequency to determine statistical correlation [Additional file 2]. It is important to note that, as was found for ability to grow in beer, the *bsrA, bsrB*, and *horA *genes did not demonstrate significant associations with resistance to any of the antibiotics tested, but rather with susceptibility.

When MIC was compared to ability of isolates to grow in beer, eight of the 17 antibiotics that we tested surprisingly demonstrated a significantly lower MIC in isolates that could grow in beer. The eight antibiotics included Synercid, Ampicillin, Levofloxacin, Penicillin, Ciprofloxacin, Sulfamethoxazole/Trimethoprim, Gatifloxacin, and Oxacillin + 2% NaCl. This suggests that, despite repeated exposure to antimicrobial hop-compounds in the brewery setting, *Pediococcus *isolates capable of growing in the beer tend to be more susceptible to commonly used antimicrobial compounds than are isolates which cannot grow in beer. It is possible that this association may actually be independent of the presence of hop-compounds, instead being an indication of the environment encountered within the brewery environment by the beer-spoilage isolates. Although beer-spoilage bacteria must originate from outside the brewery, isolates capable of growing in beer have presumably become highly acclimatized or especially adapted to grow in the beer environment. Ideally, beer will not contain any wild yeasts or bacteria and, as such, contaminating pediococci are growing in an environment that does not contain a plethora of antimicrobial compounds naturally created by other organisms living in the same environment. Based on this scenario, *Pediococcus *isolates entering the brewery environment from outside sources (e.g., plant materials such as hop cones or barley) would possess mechanisms of resistance against multiple antimicrobial compounds. However, upon entering the brewery environment which should be free of other competing microbes, the pediococci would encounter no selective pressures other than hop-compounds and thus fail to maintain the genetic mechanisms for antimicrobial resistance.

It is curious to note that the *bsrA *and *bsrB *genes, hop-resistance, and beer-spoilage are all significantly negatively-associated with resistance to Ciprofloxacin. Moreover, although *horA *is strongly correlated to ability to grow in beer, this gene does not show any association (negative or otherwise) with Ciprofloxacin resistance. While the underlying mechanism for this association with lowered resistance to Ciprofloxacin is unknown, it strongly suggests that hop-resistance, and in turn beer-spoilage, is linked to the presence of the *bsrA *and *bsrB *genes, while the *horA *gene may simply be correlated by chance to ability of *Pediococcus *isolates to spoil beer. That is to say, because the *bsrA *and *bsrB *genes (like the beer-spoilage phenotype) are negatively correlated to ciprofloxacin resistance, while the *horA *gene is not, the *bsrA *and *bsrB *genes are likely more closely associated with beer-spoilage than is the *horA *gene.

## Conclusion

Testing the susceptibility of *Pediococcus *isolates to antimicrobial compounds was effective using LSM in GPN3F antimicrobial susceptibility testing plates. In contrast with previous studies, we found *Pediococcus *isolates that are not intrinsically resistant to Vancomycin. A significant negative association was identified between resistance to Ciprofloxacin and the presence of the *bsrA *and *bsrB *genes as well as the hop-resistant and beer-spoilage phenotypes. Significantly lower MICs to antimicrobial compounds were found in isolates that were hop-resistant and/or capable of growing in beer. Similarly, the presence of genes previously correlated with beer-spoilage (i.e., *bsrA*, *bsrB*, and *horA*) was also found to be associated with significantly lower MICs to several of the antimicrobial compounds tested. These results suggest that the ongoing use of the antimicrobial hop-compounds in the brewing industry and the phenomenon of hop-resistance mediated by ATP-binding cassette type multi-drug transporters is not associated with the emergence of greater antimicrobial resistance in beer-spoilage pediococci.

## Methods

### Bacterial growth in beer

A list of the bacterial species tested is provided in Table 1, with the isolates comprising 29 pediococci (six species) and including six ropy (exopolysaccharide producing) strains. Speciation of bacterial strains was determined (or in the case of culture collection strains, confirmed) by sequencing of the first three variable regions of the 16S rRNA gene as previously described [4]. Parameters for induction of bacteria to grow in beer were as described by Haakensen *et al. *[4]. In brief, assessment of bacterial isolate growth in beer required adaptation of the bacteria using modified mMRS broth (MRS medium with Tween 80™ omitted [4]) supplemented with incremental concentrations of beer. Beer 1 was a filter-sterilized 4% v/v alcohol beer, pH 4.2 and averaging 9.8 bitterness units, while Beer 2 was a pasteurized 5% v/v alcohol beer, pH 3.8 and averaging 11 bitterness units. Bacteria capable of growing in either beer were considered to be beer-spoilers. Prior to testing for hop-resistance as described in Sections 2.2 and 2.3, bacteria were initially grown in 50% 2× mMRS and 50% Beer 2 as described by Haakensen *et al. *[4]. Bacteria were then grown at 30°C for 16-24 hours in 15% 2× mMRS and 85% Beer 2.

### Ability of bacteria to resist hop-compounds

All bacterial isolates were tested for resistance to hop-compounds by the hop-gradient mMRS agar plate containing ethanol method as described by Haakensen *et al. *[5]. The ability of each isolate to grow on the hop-gradient mMRS agar plate containing ethanol is provided in Additional file 2.

### Presence of beer-spoilage related genes

All bacterial isolates were tested for the presence of the putative beer-spoilage associated genes *ABC2*, *bsrA*, *bsrB*, *hitA*, *horA*, and *horC *as previously described by Haakensen *et al. *[3,4,6]. The presence or absence of these genes in each isolate is recorded in Additional file 2. Only *bsrA*, *bsrB*, and *horA *occurred with sufficient frequency for use in subsequent statistical analyses.

### Antimicrobial susceptibility testing

Antimicrobial susceptibility testing was performed using LSM and Sensititre GPN3F Gram-positive MIC plate (TREK Diagnostic Systems, Cleveland OH). Additional file 1 provides a list of antimicrobial compounds and concentration ranges tested. The GPN3F plates contained vacuum-dried antimicrobial compounds which were rehydrated when LSM containing the bacterial inoculate was added. Bacteria were diluted to approximately 10^3^-10^4 ^cfu/ml in LSM (confirmed by colony counting on MRS agar plates) and 100 μl were inoculated into each well of a Sensititre GPN3F plate. Bacteria were grown for 48 hours in a candle jar at 30°C. The MICs (μg/ml) were determined based on appearance of visible bacterial pellets in the bottom of wells.

### Statistical analysis

Non-parametric Mann-Whitney *U *(when testing for a difference between 2 independent samples) or Kruskal-Wallis *H *(in the case of > 2 independent samples) tests were used to compare the MICs for the 17 antibiotics to determine whether antibiotic resistance had an association with resistance to hops, presence of known genes associated with hop-resistance, antibiotic-resistance, as well as with the ability of *Pediococcus *isolates to grow in beer.

For some of the analyses, the indicator (categorical) variable of resistance or susceptibility to hop-compounds was created as described by Haakensen *et al. *[5]. Specifically, if a *Pediococcus *isolate was observed to have positive growth (> 3 cm) on hop-gradient agar with ethanol plates, then that isolate was categorized as 'hop-resistant'. For this indicator variable, Fisher's exact test and Spearman's correlation coefficient *ρ *were used for the comparison of gene presence and antibiotic resistance, respectively, with the hop-resistance indicator variable. All tests of significance were performed at *α *= 0.05 using SPSS Statistical Software for Windows (SPSS Inc., Chicago, IL, version 14.0).

## Authors' contributions

MH conceived the study, participated in the design, performed laboratory work, and drafted parts of the manuscript. DMV performed statistical analysis and drafted parts of the manuscript. BZ conceived the study, participated in its design and coordination, edited the manuscript, and is the holder of the research grand used to fund the study. All authors have read and approved the final manuscript.

## Supplementary Material

Additional file 1 **Range of minimum inhibitory concentrations of antimicrobial compounds summarized by species**. The data provided indicate the range of concentrations tested for each antibiotic and the range of MICs obtained for each *Pediococcus *species. Click here for file

Additional file 2 **Isolate and antibiotic MIC information**. Information regarding the isolates used in the study, and the MICs obtained for each antibiotic by each isolate. Click here for file
