# Supplementary material for: Susceptibility of Pediococcus isolates to antimicrobial compounds in relation to hop-resistance and beer-spoilage
Source: BMC Microbiol. 2009 Sep 7;9:190. doi: 10.1186/1471-2180-9-190 (PMC2746227; doi:10.1186/1471-2180-9-190)
Supplement: Additional file 1 — Range of minimum inhibitory concentrations of antimicrobial compounds summarized by species. The data provided indicate the range of concentrations tested for each antibiotic and the range of MICs obtained for each Pediococcus species. [file 1471-2180-9-190-S1.docx]

**Additional File 1 - Range of minimum inhibitory concentrations of antimicrobial compounds^a^ summarized by species.**

| **Antimicrobial**  **compound** | **Range tested**  **(μg/ml)** | **Range observed for *Pediococcus* species (N)** | | | | | | | |
| --- | --- | --- | --- | --- | --- | --- | --- | --- | --- |
|  |  | ***acidilactici*(6)** | ***claussenii*** | | ***damnosus*(1)** | ***inopinatus*(1)** | ***parvulus*** | | ***pentosaceus*(4)** |
|  |  |  | **ropy**^b^**(5)** | **non-ropy**^c^**(7)** |  |  | **ropy(1)** | **non-ropy(4)** |  |
| Ampicillin | 0.12-16 | 0.12-4 | 0.12-0.25 | 0.12-4 | 0.25 | 4 | 0.25 | 0.12-4 | 1-4 |
| Ceftriaxone | 8-64 | 8-NR^d^ | 8 | 8-NR | 8 | 8 | 8 | 8 | 8-64 |
| Ciprofloxacin | 0.5-2 | NR | 0.5-NR | 0.5-NR | 0.5 | NR | NR | 2-NR | 2-NR |
| Clindamycin | 0.12-2 | 0.12-0.5 | 0.12 | 0.12-1 | 0.12 | 0.12 | 0.12 | 0.12 | 0.12 |
| Daptomycin | 0.25-8 | 0.5-2 | 0.25-0.5 | 0.25 | 0.25 | 1 | 0.5 | 0.25-0.5 | 0.25-2 |
| Erythromycin | 0.25-4 | 0.25 | 0.25-0.5 | 0.25-4 | 0.25 | 0.25 | 0.25 | 0.25 | 0.25 |
| Gatifloxacin | 1-8 | 1-8 | 1 | 2 | 1 | 8 | 1 | 1-4 | 8-NR |
| Gentamicin^e^ | 2-16, 500 | 2-8 | 2 | 2-NR | 2 | 4 | 2 | 2 | 2-4 |
| Levofloxacin | 0.25-8 | 2-NR | 0.5-2 | 2-4 | 1 | NR | 4 | 1-NR | 4-NR |
| Linezolid | 0.5-8 | 0.5-4 | 0.5-2 | 0.5-4 | 1 | 2 | 1 | 0.5-2 | 0.5-4 |
| Oxacillin+2%NaCl | 0.25-8 | 0.25-2 | 0.25 | 0.25-4 | 0.25 | 4 | 0.25 | 0.25-NR | 0.25-8 |
| Penicillin | 0.06-8 | 0.25-NR | 0.06-0.25 | 0.06-NR | 0.06 | 2 | 0.25 | 0.06-4 | 0.5-2 |
| Rifampin | 0.5-4 | 0.5-4 | 0.12-1 | 0.5-2 | 0.5 | 2 | 2 | 0.5-4 | 0.5-4 |
| Streptomycin^f^ | 1000 | 1000 | 1000 | 1000 | 1000 | 1000 | 1000 | 1000 | 1000 |
| Synercid | 0.12-4 | 0.12-2 | 0.12-0.5 | 0.12-1 | 0.25 | 2 | 0.5 | 0.12-1 | 0.25-2 |
| Tetracycline | 2-16 | 8-NR | 2-8 | 2-NR | 2 | 16 | 16 | 2-16 | 2-NR |
| Trimethoprim/  Sulfamethoxazole^g^ | 0.5/9.5-4/76 | 0.5-NR | 0.5/9.5-NR | 0.5/9.5-NR | 0.5/9.5 | NR | 4/76 | 0.5/9.5-NR | 4/76-NR |
| Vancomycin | 1-128 | 4-NR | 16-NR | 4-NR | 16 | NR | NR | 8-NR | NR |

^a^ Dilutions were predetermined by the GPN3F antibiotic plate format.

^b^,^c^ Isolates positive and negative for exopolysaccharide rope production, respectively

^d^ NR; MIC not reached, isolate could grow at highest concentration of antibiotic tested.

^e^ A range of 2-16 μg/ml was tested as well as a concentration of 500 μg/ml.

^f^ A single concentration of 1000 μg/ml was tested.

^g^ Recorded as concentration of Trimethroprim / concentration of Sulfamethoxazole.
